# Multicentric tuberculosis at two rare sites in an immunocompetent adult

**DOI:** 10.1007/s10195-011-0157-8

**Published:** 2011-10-18

**Authors:** Saurabh Singh, Chethan Nagaraj, Ghanshyam N. Khare, Vinay Kumaraswamy

**Affiliations:** 1 Department of Orthopedics, Institute of Medical Sciences, Lanka, Varanasi, 221005 India; 2 Vikram Hospitals, No. 71/1, Millers Road, Bangalore, 560052 India

**Keywords:** Tuberculosis, Immunocompetent, Coccyx, Sternum

## Abstract

The case of a 20-year-old female who presented with refractory coccydynia and sternal pain is described. She was immunocompetent, and had no systemic features. She was diagnosed with tuberculosis of the sternal and coccygeal regions based on magnetic resonance imaging and histopathology of biopsy specimens. Conservative management with oral multidrug antituberculous therapy completely cured the patient, and she had not suffered any recurrence after three years of follow-up. This case highlights the possibility of the multicentric presentation of tuberculosis at two rare sites in the same immunocompetent patient, even though the differential diagnosis was coccydynia.

## Introduction

Tuberculosis remains one of the leading killer diseases in countries where it is endemic. There were 9 million new TB cases and approximately 2 million deaths from TB in 2004. More than 80% of all TB patients live in sub-Saharan Africa and Asia [6]. With the advent of HIV, tuberculosis is posing a serious health hazard, even in regions of the world where tuberculosis is not endemic.

The sternal and coccygeal regions are rare sites for tuberculosis, and require a high degree of suspicion for diagnosis, especially when they occur in combination in an immunocompetent patient.

## Case report

**Fig. 1:**
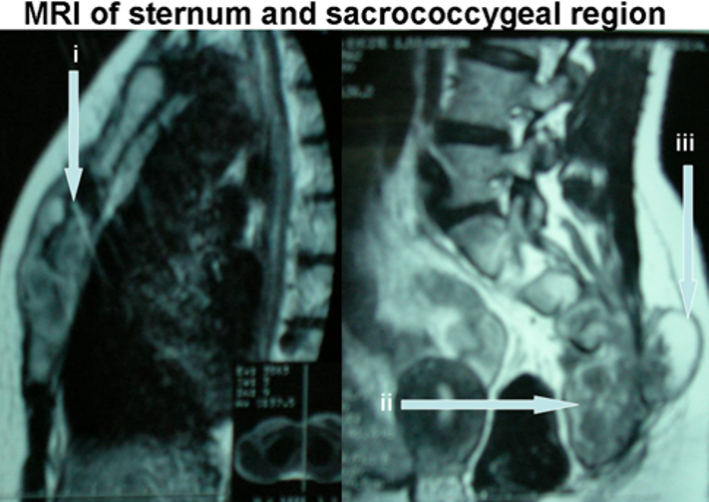
Magnetic resonance imaging of the sternal and sacrococcygeal regions. *i* Destructive lesion in the sternum, *ii* destructive lesion in the coccyx with an abscess, *iii* abscess extending into the left gluteal region

**Fig. 2:**
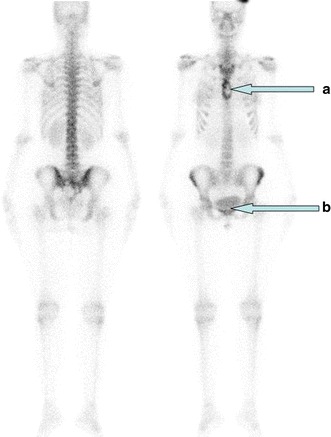
Technetium-99m MDP bone scan of the patient. *a* Photopenic area with surrounding increased tracer uptake in the sternum. *b* Photopenic area with surrounding increased tracer uptake in the coccyx

A 20-year-old female presented with a history of pain in the chest and coccygeal region for 1 year. There were no systemic symptoms of fever, weight loss, or loss of appetite. Physical examination revealed tenderness and irregularity over the sternum and tenderness over the coccyx. She was negative for HIV based on an ELISA method, and her erythrocyte sedimentation rate was 40 mm/h. Radiograph of the sternum and coccyx revealed an osteolytic lesion in the sternum and a similar lesion in the coccyx. An MRI of the sternal and sacrococcygeal regions was obtained which revealed a destructive lesion in the sternum and destruction of the coccyx with an abscess extending into the left gluteal region (Fig. 1). Technetium-99m MDP bone scintigraphy was performed, which revealed photopenic areas with mildly increased tracer uptake surrounding the photopenic areas in the sternum and coccyx, with no other bony lesions (Fig. 2).

Tru-Cut needle biopsies were obtained from the sternal and coccygeal lesions and sent for Gram staining, AFB staining, histopathology, and cultures, including a tubercular culture. The histologic picture was that of chronic inflammation with a caseating granuloma compatible with tuberculosis. A diagnosis of tuberculosis of the sternum and coccyx was established, based on the histopathology. The patient was started on antitubercular therapy in the form of a four-drug regimen consisting of rifampicin (10 mg/kg), isoniazid (10 mg/kg), pyrazinamide (35 mg/kg), and ethambutol (30 mg/kg) for 2 months, followed by rifampicin (10 mg/kg) and isoniazid (10 mg/kg) for 4 months, as per the national government’s guidelines. Along with this, local support for the coccygeal region was provided and supportive measures to improve the nutritional status were taken. The culture reports, which were obtained after 8 weeks, were positive for *Mycobacterium tuberculosis* bacilli.

The patient showed progressive improvement within 1 month of starting the therapy, and was completely relieved of her complaints after 3 months. After successfully completing the therapy for 6 months, the patient was followed up for 3 years, and showed no recurrence of symptoms.

## Discussion

Multicentric tuberculosis is usually seen in immunocompromised patients; other predisposing factors are intravenous drug use, diabetes mellitus, alcohol abuse, and hepatic cirrhosis [3]. Sternal osteomyelitis caused by *Mycobacterium tuberculosis* is a rare entity, accounting for less than 1% of all cases of osteoarticular tuberculosis, and coccygeal tuberculosis is an even rarer entity, with only one reported case in the English literature [2]. Tuberculosis at both of these sites at the same time has also not been reported in the English literature previously.

A painful coccyx is a common complaint in women due to the posterior prominence of the coccyx anatomically in the female pelvis, meaning that it is exposed to multiple traumas. However, physicians should also consider atypical conditions such as tuberculosis in patients presenting with coccygodynia, especially those who are refractory to treatment with conservative measures, and in association with HIV. Most sacral and sacrococcygeal lesions heal with adequate local support and antitubercular therapy, but coccygectomy may be essential in refractory cases.

Sternal tuberculosis usually presents as tuberculous osteomyelitis or rarely as a tuberculous granuloma, or remotely as metastatic disease [4, 5]. There have also been reports of tuberculosis of the sternum after open heart surgery. Plain radiographs are usually insufficient, and further imaging in the form of CT or MRI is usually required [1]. Most of these lesions heal with oral antitubercular therapy. However, surgical intervention in the form of resection may be required on rare occasions, followed by local rotation flaps.

Although no primary focus of infection could be located in our patient, the mode of involvement is most likely hematogenous, as suggested by its multicentric nature. Other routes of tuberculous involvement are direct inoculation, extension from adjacent bones or joints, and lymphogenous spread.

To conclude, tuberculosis can present in any form or organ of the human body. In spite of the great strides made in its management, tuberculosis continues to baffle clinicians with its varied presentations. Therefore, it is essential for all physicians to keep tuberculosis in the differential diagnosis of patients with atypical presentations in atypical locations, thus enabling its early diagnosis and better treatment.
